# Supplementary material for: Melatonin Protects Mitochondrial Function and Inhibits Oxidative Damage against the Decline of Human Oocytes Development Caused by Prolonged Cryopreservation
Source: Cells. 2022 Dec 12;11(24):4018. doi: 10.3390/cells11244018 (PMC9776420; doi:10.3390/cells11244018)
Supplement: Supplementary file 1 [file cells-11-04018-s001.zip › cells-2014007-supplementary.pdf]

Supplementary Data

Supplementary Materials and Methods

Chemicals and reagents

The oocytes and embryo culture (including fertilization, cleavage, and blastocyst stages) medium were purchased from COOK Co. (Bloomington, IN, USA). Recombinant human follicle-stimulating hormone (r-hFSH) was purchased from Merck Serono Co. (Geneva, Switzerland). A gonadotropin-releasing hormone antagonist was purchased from Ipsen Pharma (Boulogne-Billancourt, France). Human chorionic gonadotropin (HCG) was purchased from Livzon Pharmaceutical Group, Inc. (Zhuhai, China). The reactive oxygen species (ROS) assay kit was purchased from Beyotime Biotechnology Inc. (Shang Hai, China). Cell Tracker™ Fluorescent Probes and BODIPY™ FL were purchased from Thermo Fisher Scientific (Waltham, MA, USA). Finally, 4% paraformaldehyde was purchased from Moore Biotechnology Co., LTD. (Wuhan, China).

Immature oocyte collection

The immature oocyte collection protocol was performed as described previously[1]. Briefly, a total of 253 infertile women under 35 years old undergoing intracytoplasmic single sperm injection (ICSI) treatment were recruited. The discarded immature oocytes were collected (vesicula germinativa) during controlled ovarian hyperovulation (COH) cycles. All the patients in this study were treated with a gonadotropin hormone-releasing hormone (GnRH) antagonist (flexible regimen) for ovarian stimulation. Recombinant human follicle-stimulating hormone (Gn, Gonal F; Serino Barueri, SP, Brazil) was given on the second or third day of the menstrual cycle for ovarian hyperstimulation. After four to five days, the dosage of Gn was adjusted according to the follicle size and hormone level. When the dominant follicle reached 12-14 mm, GnRH antagonist (GnRH-A, Cetrotide,

Merck Serono SA-Geneva, Switzerland) was added until the day of human chorionic gonadotropin (HCG) injection. When there were two or three follicles with a diameter ≥18 mm, HCG (10,000 U; Pregnyl; AESCA Pharma, Austria) was injected, and oocyte retrieval was completed after 36-38 h under the guidance of a transvaginal ultrasound. Morphological grading was performed on oocytes under the microscope; germinal vesicle (GV) and metaphase I (MI) are immature oocytes.

In vitro maturation (IVM)

The detailed process of IVM has been previously reported in the published literature[2]. In brief, the collected immature oocytes were washed twice in the gamete medium and placed in the IVM medium, prepared and balanced overnight (50 mL per droplet of 5 oocytes under mineral oil), and cultured in a humidified atmosphere of 6% CO2 and 5% O_2 at 37 °C for 24 hours. Then, the maturity of oocytes was evaluated under an inverted microscope (IX-71; Olympus, Japan). Subsequently, the IVM-MII oocytes were selected for ICSI insemination, followed by five or six days of embryo culture *in vitro*. In total, 990 immature oocytes from 253 infertile patients were collected, and 595 (60.10%) IVM-MII oocytes were obtained by IVM culture for this study.

Preparation of the vitrification and warming media

The general vitrification and warming media used in this study were prepared as previously described[3]. In brief, the vitrification media contained equilibration medium (EM) [30% (v/v) SPS, HTF1024, 7.5% (v/v) ethylene glycol (EG), and 7.5% (v/v) 1,2-propanediol (PrOH)] and vitrification medium (VS) [HTF1024, 15% (v/v) PrOH, 30% (v/v) SPS, 15% (v/v) EG, and 0.5 M trehalose]. The warming media contained thawing medium (TM) (HTF1024, 30% SPS, and 1.0 M trehalose), diluent medium I (DM-I) (HTF1024, 30% SPS, and 0.5 M trehalose), DM-II (HTF1024, 30% SPS, and 0.25 M trehalose), and washing medium (30% SPS and HTF1024). Melatonin was dissolved with anhydrous ethanol and added to the vitrification and heating media to yield a

final concentration of 10^{-9}mol/L. These media were preheated at 37 °C for half an hour prior to each vitrification and warming procedure.

Oocyte vitrification and warming

The detailed process of vitrification and warming was described in the previously published literature[3]. Oocytes were cryopreserved with a vitrification kit (Kitazato, Japan). The IVM-MII oocytes were placed in EM solution, balanced at room temperature for 10 minutes, and then transferred into VS solution. After mixing for 60 seconds, the oocytes were placed on Cryotops (Kitazato, Japan) and plunged into liquid nitrogen. During the warming procedure, after the cryopreservation of oocytes for different times, the cryotops were removed from the liquid nitrogen and placed in pre-warmed TM solution for one minute. Then, the oocytes were sequentially transferred to DM-1, DM-2, and WS solutions at 37 °C, and the time was controlled at 3, 5, and 3 min, respectively. Afterward, the oocytes were transferred to gamete insemination fluid (COOK, USA), and then placed in a 6% CO_2, 37 °C incubator for 2.5 hours before ICSI insemination or detection.

ICSI and Embryo culture protocols

A total of 280 IVM-MII oocytes from 140 young infertile women (< 35 years old) were collected and randomly assigned to the following five groups: F group (n=50), nMC-0 group (n=58), MC-0 group (n=60), nMC-6 group (n=55) and MC-6 group (n=57), and then subjected to ICSI insemination with the donated sperm. There was no significant difference in the baseline data of the patients among these groups (Table S1). The injected oocytes were individually cultured in Time Lapse at 37 °C with 6% CO_2 and 5% O_2. The embryo culture protocol was performed as described previously[1]. In brief, the oocytes undergoing *in vitro* culture in the balanced cleavage culture droplets (one oocyte/drop) were observed based on their fertilization status 14-16 h after insemination, and then the culture was continued at 37 °C, 6% CO_2, and saturated humidity. Two days later, all of the formed cleavage

embryos were transferred into balanced blastocyte culture droplets (one to three embryos/drop) for an additional two or three days of blastocyst culture. The number of viable oocytes, fertilized oocytes, cleavage-stage embryos, high-quality cleavage-stage embryos, and blastocysts was examined and reported, as previously[2]. In this process, all the embryos formed were scored according to the Tomas[4] and Gardner[5] scoring standards.

Single-oocyte proteomic assay

Overall, 3 oocytes in each group were prepared for single-oocyte proteomic analysis. Three parallel experiments were conducted for each group. Oocytes under five different conditions were washed three times in fertilization medium. The protease inhibitor cocktail was diluted according to 1RU 100 with PBS, and the mixture of 3ul in each PCR tube was packed separately. The collected high-quality IVM-MII oocytes were placed in the prepared PCR tube with 3 oocytes in each group, one for each tube and stored at -80 ℃. Lysis buffer was added to the sample, and the sample was lysed by sonication, followed by reduction and alkylation, and then trypsin digestion. Samples were separated using the Thermo UltiMate 3000 UHPLC. The sample first entered the trap column for enrichment and desalting, and was then connected in series with a self-packed C18 column and separated at a flow rate of 500 nl / min. The peptides separated by the liquid phase were ionized by the nanoESI source and entered the tandem mass spectrometer Orbitrap Fusio™ Lumos™ Tribrid™ (Thermo Fisher Scientific, San Jose, CA) for DDA (data-dependent acquisition) mode detection. The micro-sample data and the library sample data of MS were used with MaxQuant's integrated Andromeda engine and "match between runs" mode to complete the identification. Then, MaxQuant used the peptide peak intensity, peak area, and LC retention time related to MS1 and other information to analyze the obtained data. We performed a series of statistical analyses and quality control on micro-samples, and then completed GO, COG, pathway, and other functional annotation

analyses based on the protein identification results; we then performed a series of statistical analyses on the library samples. According to the set of comparison groups, the multiple differences in the proteins in each comparison group were calculated, and the significance test was performed using Welch's t-test. Furthermore, screening was performed based on the multiple difference >1.5 and *P*-value <0.05 as the criteria for determining significant differences.

Detection of ATP levels in oocytes

A total of 94 IVM-MII oocytes collected from 37 young infertile women (<35 years) who received ICSI were randomly assigned to the following five groups:the nMC-0 group (n=20), the nMC-6 group (n=17), the MC-0 group (n=17), the MC-6 group (n=17) and the F group (n=23). There was no significant difference in the baseline data of the patients among these groups (Table S2). ATP production in 94 high-quality IVM-MII oocytes was evaluated with the BODIPY™ FL ATP kit (A12410, Invitrogen). Briefly, the examined oocytes were fixed in 4% paraformaldehyde for 1 hour, washed three times, and incubated in PBS supplemented with 500 nM BODIPY™ FL ATP for 1 h at room temperature in the dark. Then, oocytes were washed 3 times in PBS, mounted on glass substrate dishes and observed under a laser scanning confocal microscope (LSM 800, Zeiss, Germany). The fluorescence intensity of each oocyte was analyzed using ImageJ software (Research Services Branch, National Institute of Mental Health).

Detection of ROS levels in oocytes

A total of 84 IVM-MII oocytes collected from 37 young infertile women (<35 years) who received ICSI were randomly assigned to the following five groups:the nMC-0 group (n=17), the nMC-6 group (n=17), the MC-0 group (n=16), the MC-6 group (n=15) and the F group (n=19). There was no significant difference in the baseline data of the patients among these groups (Table S3). These oocytes of each groups were loaded with the

oxidation-sensitive fluorescent probe DCFH-DA (S0033S, Beyotime Biotechnology co., Ltd.) and incubated at 37°C for 30 min in the dark. Afterward, the oocytes were washed three times with PBS and placed on glass substrate dishes. The fluorescent intensity of each oocyte was assessed using the laser scanning confocal microscope and analyzed using ImageJ software.

Detection of GSH levels in oocytes

A total of 86 IVM-MII oocytes collected from 36 young infertile women (<35 years) who received ICSI were randomly assigned to the following five groups:the nMC-0 group (n=17), the nMC-6 group (n=17), the MC-0 group (n=16), the MC-6 group (n=16) and the F group (n=20). There was no significant difference in the baseline data of the patients among these groups (Table S4). GSH levels in 86 IVM-MII oocytes were measured with the CellTracker™ Fluorescent Probe (C12881, ThermoFisher Scientific). In brief, the examined oocytes in each group were stained with 10 μM CellTracker™ at 37 °C for 30 min and then washed with PBS to eliminate the surface fluorescence. Fluorescence intensity was measured by a laser scanning confocal microscope. The photographs were analyzed using the ImageJ software to measure the staining brightness of each oocyte.

Supplementary Figure

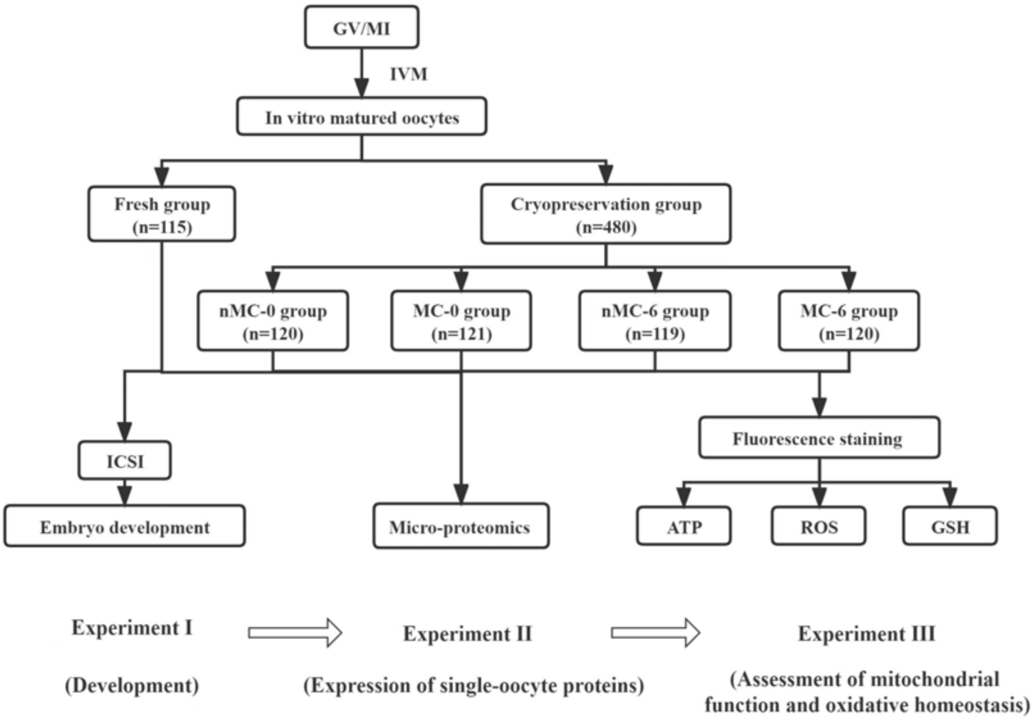

Figure S1. A flowchart of the experimental design.

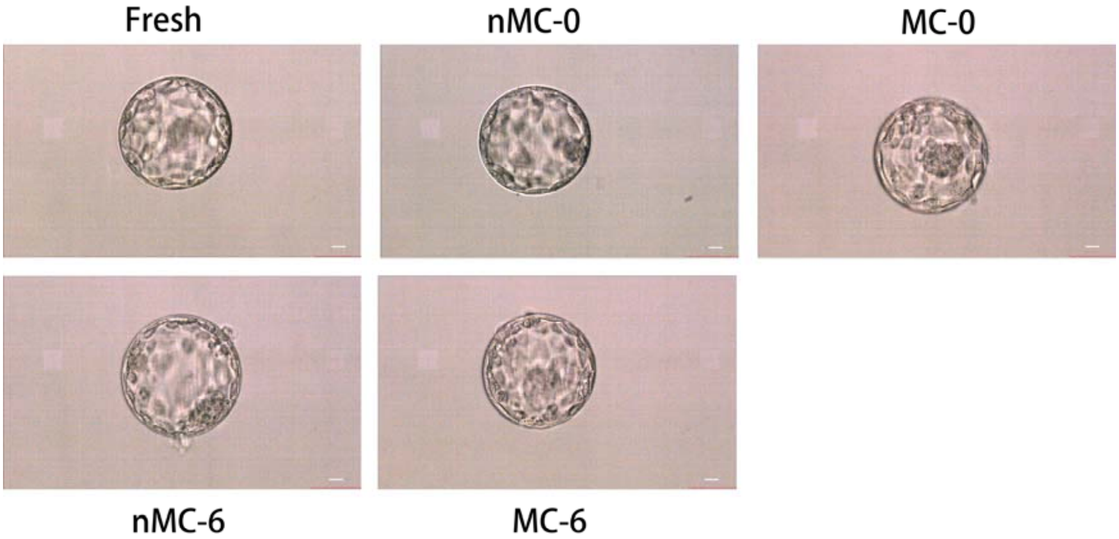

Figure S2. Representative images of the high-quality blastocysts. (1) Fresh group: high-quality blastocyst 4AA; (2) nMC-0 group: high-quality blastocyst 4AB; (3) MC-0 group: high-quality blastocyst 4AA; (4) nMC-6 group: high-quality blastocyst 4BB; (5) MC-6 group: high-quality blastocyst 4AB. Bar = 10 μm.

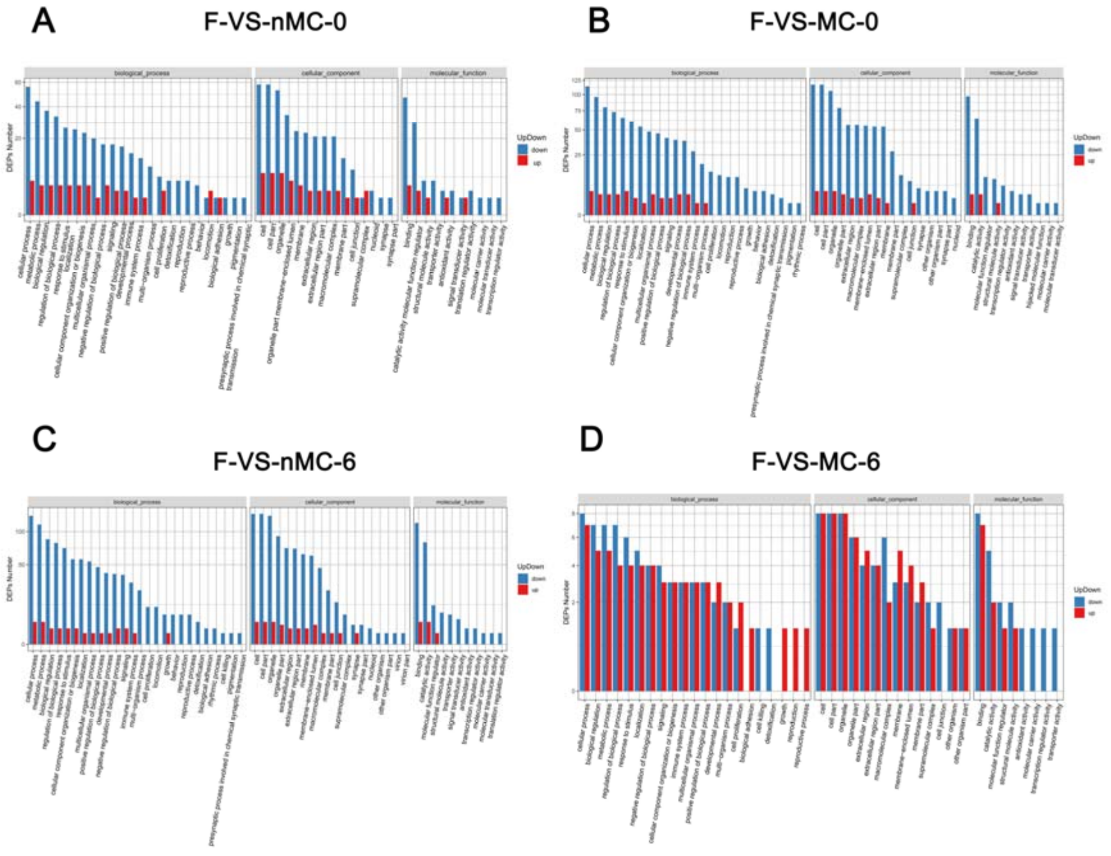

Figure S3. Differential protein GO function classification map of cryopreserved human oocytes from the nMC-0 group, MC-0 group, nMC-6 group and MC-6 group. Fresh oocytes (F group) served as the control. (A) F VS nMC-0: Comparison of up-and down-regulated differential proteins between group F and group nMC-0; (B) F VS MC-0: Comparison of up-and down-regulated differential proteins between group F and group MC-0; (C) F VS nMC-6: Comparison of up-and down-regulated differential proteins between group F and group nMC-6; (D) F VS MC-6: Comparison of up-and down-regulated differential proteins between group F and group MC-6. The red represents up, the blue represents down.

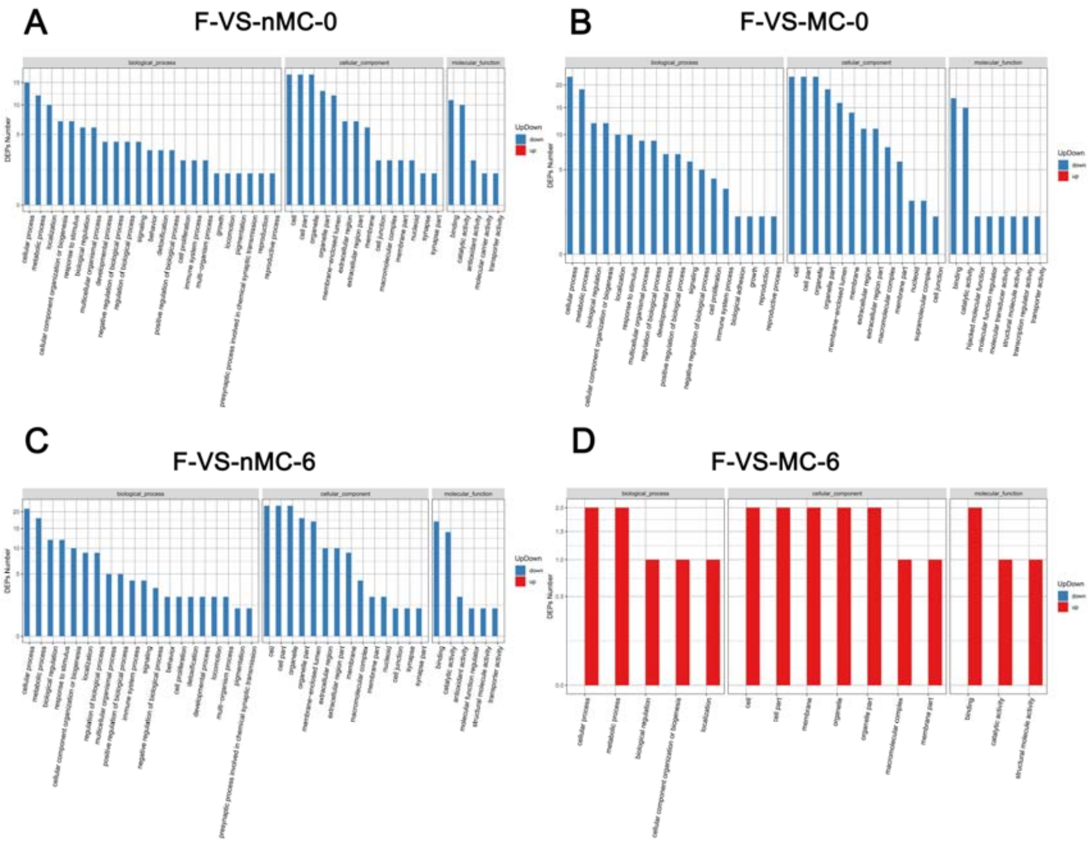

Figure S4. GO functional classification map of differential proteins associated to mitochondrial of cryopreserved human oocytes from the nMC-0 group, MC-0 group, nMC-6 group and MC-6 group. Fresh oocytes (F group) served as the control. (A) F VS nMC-0: Comparison of up-and down-regulated differential proteins between group F and group nMC-0; (B) F VS MC-0: Comparison of up-and down-regulated differential proteins between group F and group MC-0; (C) F VS nMC-6: Comparison of up-and down-regulated differential proteins between group F and group nMC-6; (D) F VS MC-6: Comparison of up-and down-regulated differential proteins between group F and group MC-6. The red represents up, the blue represents down.

Supplementary Table

Table S1. Comparison of baseline data of the 5 groups of patients in the developmental competence.

	F	nMC-0	MC-0	nMC-6	MC-6	*P*- value
Female age (yr)	30.32±3.611	29.46±4.096	30.32±3.151	31.00±2.708	29.29±3.599	ns
Infertility period (yr)	2.696±1.012	2.786±1.228	3.143±1.353	3.286±1.301	2.821±1.090	ns
No. of retrieved oocytes	17.21±5.600	18.00±6.377	16.96±6.028	18.11±7.015	17.89±5.350	ns
FSH, mIU/ml	5.848±2.669	6.224±2.927	5.564±2.511	5.824±2.899	4.919±2.617	ns
LH, mIU/ml	4.451±2.471	5.038±2.408	4.610±2.418	5.721±2.509	5.967±2.431	ns
E2, mIU/ml	254.1±420.3	259.2±217.6	239.6±288.8	204.9±331.3	201.8±182.4	ns
BMI, mIU/ml	21.58±2.845	21.81±2.852	21.65±2.351	22.65±3.420	22.84±2.603	ns

Datas analyzed using one-way ANOVA. Values are presented as mean ± standard deviation.

Table S2. Comparison of baseline data of the 5 groups of patients in the the detection of ATP.

	F	nMC-0	MC-0	nMC-6	MC-6	*P*- value
Female age (yr)	31.00±3.559	30.86±3.288	29.29±4.645	29.25±2.964	27.29±3.147	ns
Infertility period (yr)	3.429±1.272	3.714±1.254	2.714±1.380	3.125±0.991	2.571±1.397	ns
No. of retrieved oocytes	17.57±4.467	19.86±5.900	21.71±3.729	15.88±6.937	17.71±4.152	ns
FSH, mIU/ml	6.763±2805	5.614±3.072	5.190±2.341	5.955±2.601	6.576±2.037	ns
LH, mIU/ml	5.247±1.600	2.849±1.856	5.853±1.871	4.970±2.314	4.521±2.300	ns
E2, mIU/ml	226.6±288.1	139.0±96.51	459.4±790.2	190.6±83.31	248.6±199.8	ns
BMI, mIU/ml	21.65±2.230	22.99±3.345	21.47±1.556	20.39±3.417	22.76±3.254	ns

Datas analyzed using one-way ANOVA. Values are presented as mean ± standard deviation.

Table S3. Comparison of baseline data of the 5 groups of patients in the detection of ROS.

	F	nMC-0	MC-0	nMC-6	MC-6	*P*-value
Female age (yr)	31.29±2.563	31.50±2.777	30.00±2.507	30.50±4.680	28.57±3.690	ns
Infertility period (yr)	2.714±1.113	2.500±0.926	3.000±1.309	2.250±0.758	3.000±1.265	ns
No. of retrieved oocytes	17.57±7.115	16.25±5.339	17.13±5.643	17.67±4.926	20.75±7.592	ns
FSH, mIU/ml	4.047±3.310	6.223±2.406	5.774±2.642	4.732±1.576	5.353±2.941	ns
LH, mIU/ml	4.153±1.148	3.766±2.522	5.130±3.086	4.512±2.854	8.356±3.510	ns
E2, mIU/ml	189.7±214.8	148.1±134.8	159.1±259.7	436.2±600.7	107.6±154.7	ns
BMI, mIU/ml	23.06±3.469	21.66±1.805	21.44±1.469	20.58±2.076	23.04±2.736	ns

Datas analyzed using one-way ANOVA. Values are presented as mean ± standard deviation.

Table S4. Comparison of baseline data of the 5 groups of patients in the detection of GSH.

	F	nMC-0	MC-0	nMC-6	MC-6	*P*-value
Female age (yr)	29.75±3.240	28.57±2.440	29.71±2.928	32.33±3.266	31.00±3.381	ns
Infertility period (yr)	2.625±1.188	2.429±0.976	2.857±1.574	3.333±0.817	2.500±1.195	ns
No. of retrieved oocytes	20.50±5.529	16.00±6.904	17.00±6.803	18.33±7.528	20.88±3.980	ns
FSH, mIU/ml	6.708±5.274	6.780±2.025	5.459±3.515	6.043±2.654	8.656±1.015	ns
LH, mIU/ml	5.033±2.810	5.566±2.342	4.564±2.392	5.105±2.219	4.556±3.214	ns
E2, mIU/ml	139.3±242.2	151.0±130.6	251.7±69.90	203.2±196.9	94.00±59.76	ns
BMI, mIU/ml	22.19±2.839	21.80±1.187	21.24±1.683	25.40±3.273	21.95±3.037	ns

Datas analyzed using one-way ANOVA. Values are presented as mean ± standard deviation.

References:

[1]. Zhu Q, Wang K, Zhang C, et al. Effect of melatonin on the clinical outcome of patients with repeated cycles after failed cycles of in vitro fertilization and intracytoplasmic sperm injection. *Zygote*. 2022. 28:1-9.

[2]. Zou H, Chen B, Ding D, et al. Melatonin promotes the development of immature oocytes from the COH cycle into healthy offspring by protecting mitochondrial function. *J Pineal Res.* 2020. 68: e12621.

[3]. Zhang Z, Wang T, Hao Y, et al. Effects of trehalose vitrification and artificial oocyte activation on the development competence of human immature oocytes. *Cryobiology*. 2017. 74:43-49.

[4]. Tomás C, Orava M, Tuomivaara L, et al. Low pregnancy rate is achieved in patients treated with intracytoplasmic sperm injection due to previous low or failed fertilization in in-vitro fertilization. *Hum Reprod*. 1998. 13(1):65-70.

[5]. Gardner DK, Schoolcraft WK. Culture and transfer of human blastocysts. *Curr Opin Obstet Gynecol*. 1999. 11:307-311.
